# Supplementary material for: A New Heart-Cutting Method for a Multiplex Quantitative Analysis of Steroid Hormones in Plasma Using 2D-LC/MS/MS Technique
Source: Molecules. 2023 Feb 1;28(3):1379. doi: 10.3390/molecules28031379 (PMC9921923; doi:10.3390/molecules28031379)
Supplement: Supplementary file 1 [file molecules-28-01379-s001.zip › molecules-2161006-supplementary.pdf]

Supporting Information

A New Heart-Cutting Method for a Multiplex Quantitative Analysis of Steroid Hormones in Plasma Using 2D-LC/MS/MS Technique

**Marcela Kotasova [1,*], Ondrej Lacina [2], Drahomira Springer [1], Jan Sevcik [1], Tomas Brutvan [3], Jana Jezkova [3]
and Tomas Zima [1]**

[1] Institute of Medical Biochemistry and Laboratory Diagnostics, General University Hospital, The First Faculty of Medicine of Charles University, 128 00 Prague, Czech Republic
[2] Department of Food Analysis and Nutrition, University of Chemistry and Technology, 16628 Prague, Czech Republic
[3] 3rd Department of Internal Medicine—Endocrinology and Metabolism, General University Hospital, The First Faculty of Medicine of Charles University, 12800 Prague, Czech Republic
* Correspondence: marcela.kotasova@vfn.cz

* Corresponding author. Email address: marcela.kotasova@vfn.cz (M. Kotasová)

Supporting information includes 5 tables, 3 figuers, and 7 pages.

Table S1. Mobile phase gradients used in the 1st dimension column (a) and in the 2nd dimension column (b) for the steroid hormones method.

a)

HILIC Column			
Time (min)	A1 (%)	B1 (%)	Flow (mL/min)
0.00	10	90	0.3
2.00	10	90	0.3
2.10	80	20	0.4
7.00	80	20	0.4
7.10	0	100	0.45
13.00	0	100	0.45
13.10	50	50	0.3
14.50	50	50	0.3
14.60	10	90	0.3

b)

RP Column			
Time (min)	A (%)	B (%)	Flow (mL/min)
0.00	90	10	0.5
0.80	90	10	0.5
0.83	100	0	2.0
1.31	100	0	2.0
1.34	100	0	0.5
1.50	55	45	0.5
10.00	45	55	0.5
14.00	0	100	0.5
16.00	0	100	0.7
16.10	90	10	0.5

Table S2. Valve position during analysis for steroid hormones method.

Valve 1	
Time (min)	Position
0.00	2A
0.80	1B
1.35	2A
2.90	1B
17.00	2A

Valve 2	
Time (min)	Position
0.00	2A
0.80	1B
1.35	2A

Table S3. Mobile phase gradients used in the 1st dimension column (a) and in the 2nd dimension column (b) for estradiol independent analytical run (IAR).

a)

HILIC Column			
Time (min)	A1 (%)	B1 (%)	Flow (mL/min)
0.00	10	90	0.3
1.50	10	90	0.3
1.60	80	20	0.4
2.50	80	20	0.4
2.60	0	100	0.4
6.00	0	100	0.4
6.10	50	50	0.4
6.50	50	50	0.4
6.60	10	90	0.4

b)

RP Column			
Time (min)	A (%)	B (%)	Flow (mL/min)
0.00	90	10	0.5
0.80	90	10	0.5
0.83	100	0	2.0
1.32	100	0	2.0
1.35	100	0	0.5
1.40	30	70	0.5
6.00	0	100	0.5
8.00	0	100	0.6
8.10	90	10	0.6

Figure S1. Gradients and flow rates of the two-dimensional method for estradiol IAR.

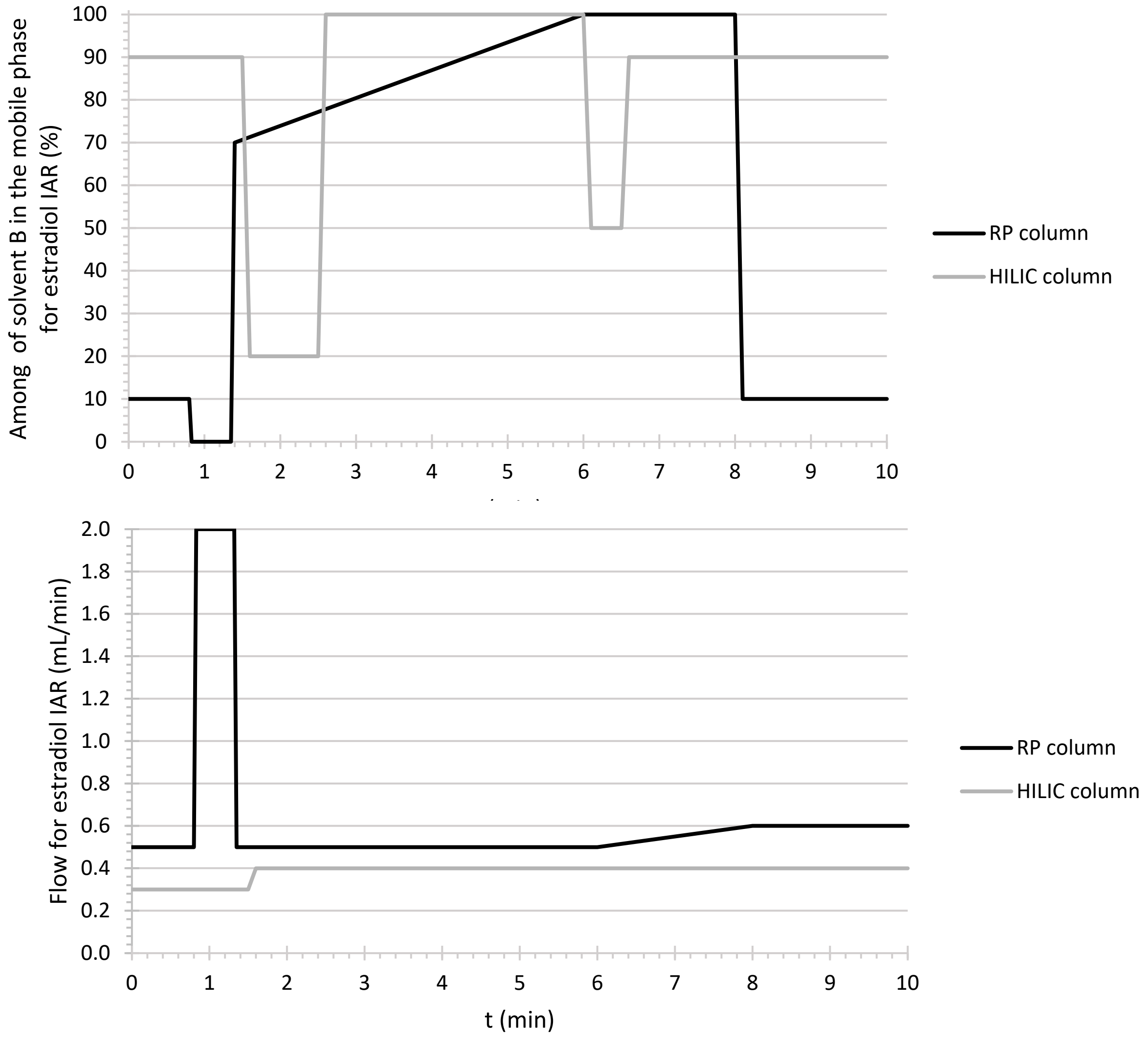

Table S4. Valve position during analysis for estradiol IAR.

Valve 1	
Time (min)	Position
0.00	2A
0.80	1B
1.35	2A
2.00	1B
9.00	2A

Valve 2	
Time (min)	Position
0.00	2A
0.80	1B
1.35	2A

Figure S2. Valve position during analysis for estradiol IAR.

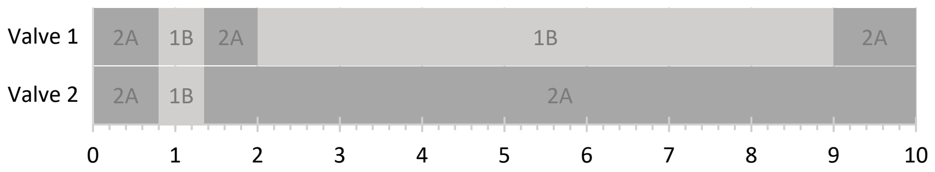

Figure S3. Settings of valves position and flow of mobile phases in position (a) Valve 1-1B/Valve 2-1B -sample is injected to system into extraction column, analytes are separated from matrix and retained at TRAP, (b) Valve 1-2A/Valve 2-2A - analytes are eluted to analytical column (RP) and analyzed in MS system, (c) Valve 1-1B/Valve 2-2A - analyzes is running and trap is washing out from the opposite side.

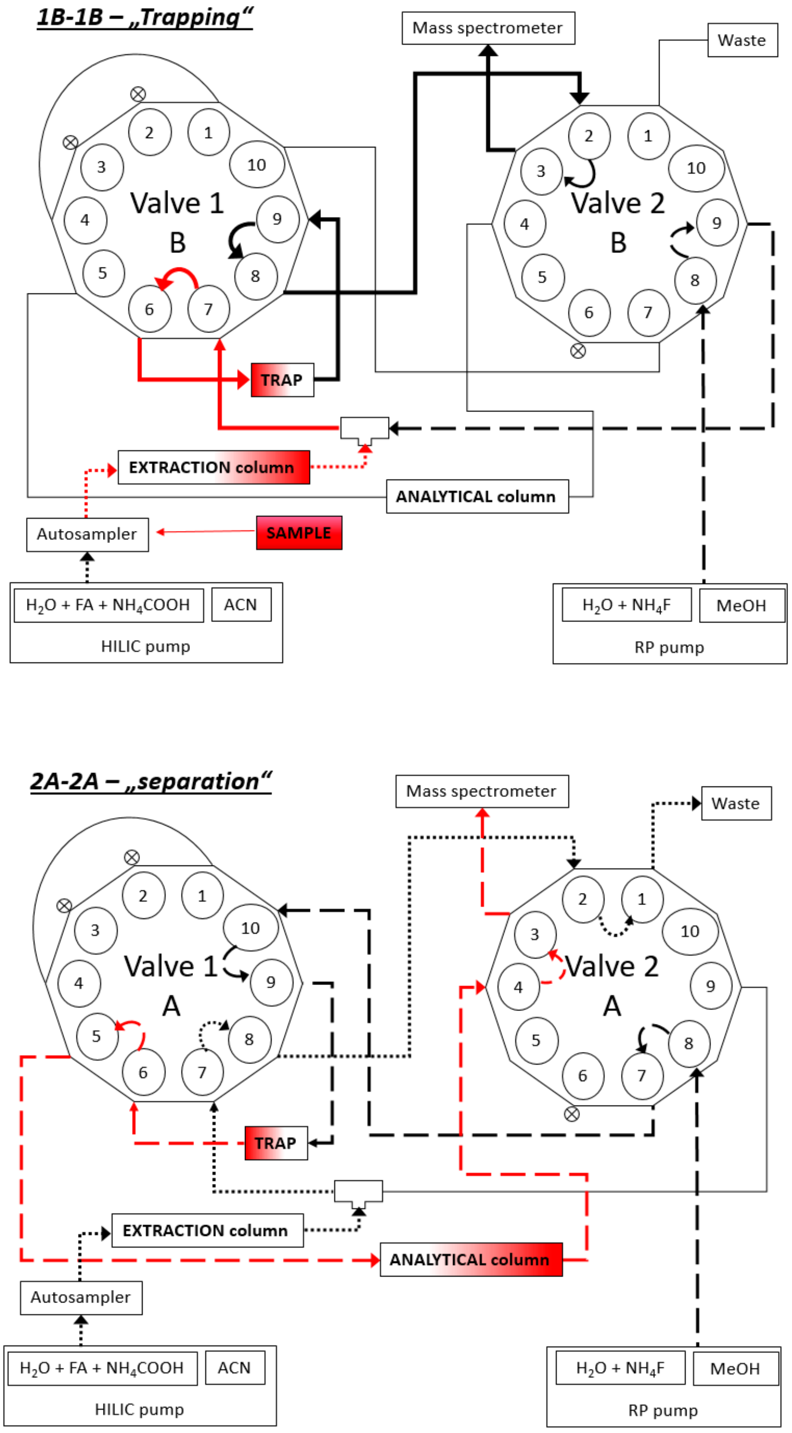

1B-2A – „washing“

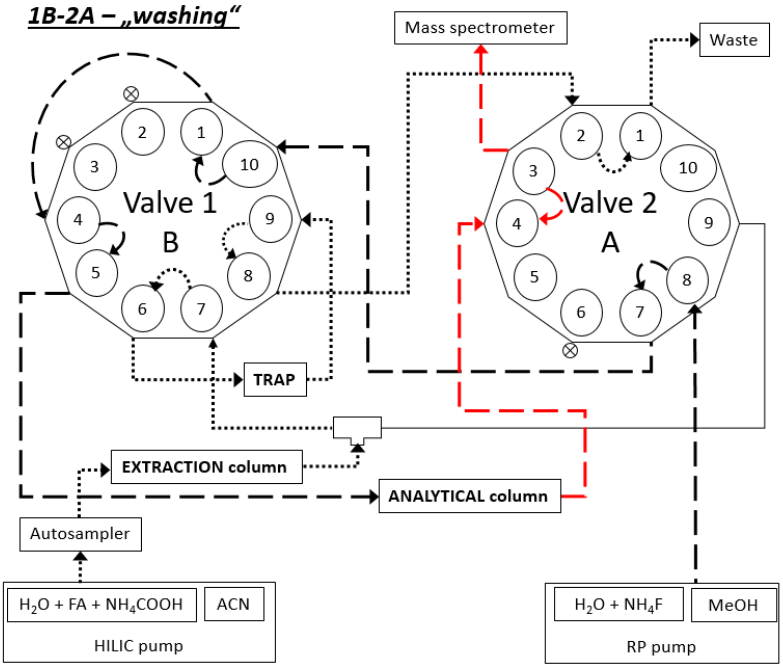

Table S5. Mass transmission, MRM condition, and retention time for each hormone and their label internal standard.

Compound Name	Precursor Ion (m/z)	Product Ion (m/z)	Fragmentor (V)	Cell voltage (V)	Retention Time (min)
11-deoxycorticosterone	331.2	109.1	102	28	12.4
11-deoxycorticosterone-D8	339.3	113.1	126	24	12.3
11-deoxycortisol	347.2	97.1	129	32	10.6
11-deoxycortisol-D5	352	100.1	141	28	10.5
17α-hydroxyprogesterone	331.2	109.1	121	31	12.8
17α-hydroxyprogesteron-13C3	334.2	100.1	117	28	12.9
21-deoxycortisol	347.2	121.1	123	16	9.5
21-deoxycortisol-D8	355.2	125.0	132	16	9.4
Aldosterone	361.2	343.2	132	20	6.1
Aldosterone-D4	365.2	346.2	140	20	6.1
Androstenedione	287.2	97.1	135	28	11.8
Androstenedione-13C3	290.2	100.1	123	24	11.8
Corticosterone	347.2	121.1	141	16	10.2
Corticosterone-D8	355.2	125.0	168	16	10.0
Cortisol	363.2	121.1	135	24	7.8
Cortisol-D4	367.2	121.1	141	28	7.8
Cortisone	361.2	163.1	144	28	6.9
Cortisone-D8	369.2	168	111	26	6.9
DHEA	289.1	213.1	95	12	12.8
DHEA-D5	294.1	218.1	95	16	12.8
DHEAS	367.2	96.9	165	60	8.3
DHEAS-D6	373.2	97.0	165	60	8.3
Dihydrotestosterone	291.2	255.1	120	16	13.4
Dihydrotestosterone-D3	294.1	258.1	123	16	13.4
Estradiol	271.2	183.1	165	48	12.5
Estradiol- D5	276.2	187.0	168	52	12.2
Estradiol IAR	271.2	183.1	165	48	4.0
Estradiol- D5 IAR	276.2	187.0	168	52	4.0
Progesterone	315.2	109.1	112	28	13.7
Progesterone-13C3	318.3	112.1	110	28	13.7
Testosterone	289.2	97.1	121	25	12.5
Testosterone-D3	292.1	97.1	121	28	12.5
